# Infectious SARS-CoV-2 Particles from Rectal Swab Samples from COVID-19 Patients in Brazil

**DOI:** 10.3390/v15051152

**Published:** 2023-05-11

**Authors:** Ieda Pereira Ribeiro, Lilian Gonçalves do Nascimento, Luis Fernando Lopez Tort, Elisa Cavalcante Pereira, Lidiane Souza Raphael Menezes, Fabio Correia Malta, Barbara Cristina Euzebio Pereira Dias de Oliveira, João Paulo Rodrigues, Pedro Paulo de Abreu Manso, Marcelo Pelajo, Myrna Cristina Bonaldo, Paola Cristina Resende Silva, Marilda Mendonça Siqueira, Patricia Brasil, Tulio Machado Fumian

**Affiliations:** 1 Laboratório de Medicina Experimental e Saúde, Instituto Oswaldo Cruz, Fundação Oswaldo Cruz, Rio de Janeiro 21040-900, RJ, Brazil; ieda.ribeiro@ioc.fiocruz.br (I.P.R.);; 2 Laboratório de Virologia Comparada e Ambiental, Instituto Oswaldo Cruz, Fundação Oswaldo Cruz, Rio de Janeiro 21040-900, RJ, Brazil; 3 Laboratório de Vírus Respiratórios, Exantemáticos, Enterovírus e Emergências Virais, Instituto Oswaldo Cruz, Fundação Oswaldo Cruz, Rio de Janeiro 21040-900, RJ, Brazil; 4 Laboratório de Virologia Molecular, Universidad de la República, Centro Universitario Regional Litoral Norte, Salto 50000, Uruguay; 5 Laboratório de Doenças Febris Agudas, Instituto Nacional de Infectologia Evandro Chagas, Fundação Oswaldo Cruz, Rio de Janeiro 21040-900, RJ, Brazil

**Keywords:** SARS-CoV-2, acute gastroenteritis, children, young adults, stool samples, Brazil

## Abstract

The main objective of this study was to investigate the dynamic of SARS-CoV-2 viral excretion in rectal swab (RS), saliva, and nasopharyngeal swab (NS) samples from symptomatic patients and asymptomatic contacts. In addition, in order to evaluate the replication potential of SARS-CoV-2 in the gastrointestinal (GI) tract and the excretion of infectious SARS-CoV-2 from feces, we investigated the presence of subgenomic nucleoprotein gene (N) mRNA (sgN) in RS samples and cytopathic effects in Vero cell culture. A prospective cohort study was performed to collect samples from symptomatic patients and contacts in Rio de Janeiro, Brazil, from May to October 2020. One hundred and seventy-six patients had samples collected at home visits and/or during the follow up, resulting in a total of 1633 RS, saliva, or NS samples. SARS-CoV-2 RNA was detected in 130 (73.9%) patients who had at least one sample that tested positive for SARS-CoV-2. The presence of replicating SARS-CoV-2 in RS samples, measured by the detection of sgN mRNA, was successfully achieved in 19.4% (6/31) of samples, whilst infectious SARS-CoV-2, measured by the generation of cytopathic effects in cell culture, was identified in only one RS sample. Although rare, our results demonstrated the replication capacity of SARS-CoV-2 in the GI tract, and infectious viruses in one RS sample. There is still a gap in the knowledge regarding SARS-CoV-2 fecal–oral transmission. Additional studies are warranted to investigate fecal or wastewater exposure as a risk factor for transmission in human populations.

## 1. Introduction

The ongoing pandemic of coronavirus disease 2019 (COVID-19), caused by severe acute respiratory syndrome coronavirus 2 (SARS-CoV-2), has had an unprecedented effect on public health systems and economies worldwide [1]. As of 11 March 2023, over 760 million confirmed cases and almost 7 million deaths were reported (covid19.who.int). SARS-CoV-2 is a positive-sense single-stranded RNA virus, within the family *Coronaviridae*, genus *Betacoronavirus* [2]. Coronaviruses are usually related to respiratory and intestinal infections in animals and humans [3]. SARS-CoV-2 is an airborne human pathogen transmitted by droplets and aerosols [4,5]. The symptoms of SARS-CoV-2 infection can vary widely. Most cases present as a classical respiratory disease, but COVID-19 can include gastrointestinal, neurological, renal, and cardiovascular symptoms and other complications [6,7,8]. SARS-CoV-2 cellular receptor (angiotensin-converting enzyme 2—ACE2) is highly expressed in lung cells and gut enterocytes [9]. The infection of proximal and distal cells in the gut is linked to gastrointestinal symptoms, such as nausea and diarrhea, frequently reported by COVID-19 patients [10,11]. In addition to the presence of ACE2 in cell types of both respiratory and intestinal tracts, the gut–lung axis shares common exposure to external microbes via the oral route [12]. This constant exposure shapes both tract microbiomes that may influence susceptibility to respiratory disorders and different inflammatory responses among COVID-19 patients [13,14,15].

Although a few studies have detected infectious SARS-CoV-2 in stool or urine samples [16,17], the role of fecal–oral transmission of SARS-CoV-2 and environment-to-human COVID-19 spread is still unclear [18]. Approximately half of the patients affected by COVID-19 developed gastrointestinal symptoms such as diarrhea, nausea, and abdominal pain, with several studies reporting the presence of SARS-CoV-2 in stool samples from patients with or without diarrhea-associated symptoms [19,20,21]. A review study investigating potential intestinal infection and fecal–oral transmission reported a detection rate of SARS-CoV-2 RNA varying from 15.3% to 81.8% in stool and rectal swab samples [22]. In addition, SARS-CoV-2 RNA has been detected in stool for up to a month in patients with mild infection [23,24]. More recently, Cerrada-Romero et al. [25] demonstrated SARS-CoV-2 RNA detection in stool samples of almost half of adult COVID-19 patients; however, replicating virus was not found in any of the stool samples.

Although many studies have demonstrated the detection of SARS-CoV-2 RNA in stool and rectal swab samples by molecular methods (reviewed by Guo et al. [22]), there is scant data on viral infectivity in these samples. Identifying possible alternative routes of infection, in addition to the airborne route, is crucial to understand SARS-CoV-2 pathogenesis and to develop future public health prevention strategies. Therefore, we investigated the presence of infectious SARS-CoV-2 in rectal swab samples collected from COVID-19 patients using Vero cell lines. In addition, we also searched for subgenomic RNA to confirm the virus’ ability to replicate in the gastrointestinal tract.

## 2. Materials and Methods

### 2.1. Sample Collection, Subjects, and Study Design

Nasopharyngeal swab (NS), saliva, and rectal swab (RS) samples were obtained from a major study that aimed to investigate SARS-CoV-2 dynamics among patients and household contacts in a slum in Rio de Janeiro, Brazil [26]. We collected and analyzed 1633 samples from 176 symptomatic individuals and contacts, including children, adolescents, and adults, from May to October 2020. Samples were collected during home visits as described elsewhere [26].

From this total, we collected and analyzed 697 NS, 603 saliva, and 333 RS samples, with a median of 4 NS and saliva samples per participant. NS and saliva samples were used to screen COVID-19-positive patients by real-time RT-PCR targeting the E gene and the RdRp region of the Orf1ab gene of SARS-CoV-2 using the Charité protocol [27].

This present study is primarily focused on RS samples obtained from the participants. We analyzed 333 RS samples, with a median of two samples (ranging from 1 to 5) per individual, collected during home visits using a sterile cotton swab and immersed in sterile tubes containing viral transport media (VTM).

### 2.2. Ethics Statements

This study was approved by the Ethics Committee of the Oswaldo Cruz Foundation (FIOCRUZ), number CAAE: 30639420.0.0000.5262, and written informed consent was obtained from the participants.

### 2.3. SARS-CoV-2 Genomic and Subgenomic RNA Detection and Quantification

SARS-CoV-2 RNA from RS samples was detected and quantified using a quantitative one-step RT-PCR (RT-qPCR) with N1 and N2 primers and probe sets (Integrated DNA Technologies, Coralville, IA, USA. N1 and N2 assays were designed to target different regions within the 5′, middle, and 3′ regions of the N gene, and both demonstrated high sensitivity and specificity for detecting up to five RNA copies/reactions, as previously described [28]. Briefly, viral RNA was extracted from 140 μL of VTM in the QIAcube automated platform, using the QIAamp Viral RNA Mini kit (QIAGEN, Hilden, Germany). RT-qPCR was performed in a final volume of 20 μL with the SuperScript III Platinum One-Step RT-qPCR Kit (Thermo Fisher Scientific, Waltham, MA, USA) in the Applied Biosystems 7500 Real-Time PCR System (Thermo Fisher Scientific, Waltham, MA, USA) using 5 μL of purified RNA. Samples with Ct < 40 were considered positive.

To investigate potential SARS-CoV-2 replication in the gastroenteric tract, we performed RT-qPCR to identify viral subgenomic nucleoprotein gene (N) mRNA (sgN) directly in RS samples. Subgenomic transcripts were amplified using the subgenomic leader sequence of the forward primer sgLeadSARSCoV2-F: 5′-CGATCTCTTGTAGATCTGTTCTC-3′ with the reverse primer and probe for N1, as previously described [29,30].

### 2.4. SARS-CoV-2 Cell Culture Isolation

SARS-CoV-2-positive RS samples, stored at −80 °C, were processed for viral isolation in cell culture in a Biosafety Level 3 facility. Vero cells (ATCC-CCL81) were cultured at 37 °C in a humidified incubator with 5% CO_2_ in Earle’s 199 medium (Gibco, Thermo Fisher Scientific, USA) supplemented with 5% fetal bovine serum (Gibco), 0.25% sodium bicarbonate (Sigma-Aldrich, St. Louis, MO, USA) and 40 mg/mL gentamicin (Gibco) for viral passage and titration. To isolate infectious viruses, RS samples were filtered (0.22 μm, Millex-GP Syringe Filter Unit, Millipore, Burlington, MA, USA) and mixed (*v*/*v*) with maintenance Earle’s 199 culture medium including 100 U/mL of penicillin, 100 μg/mL of streptomycin, and 0.25 μg/mL of amphotericin B (Gibco, Thermo Fisher Scientific, Waltham, MA, USA). A total of 300 µL was inoculated on Vero cells seeded at 40,000 cells/cm^2^ in T-25 flasks. After 1 h of incubation with periodic shaking, 3 mL of 199 medium was added to culture cells and checked daily for cytopathic effects (CPE) under a light microscope, and viral load was determined via RT-qPCR.

Supernatant aliquots from culture cells were harvested immediately post-infection (pi), 24 h pi, 48 h pi, and 72 h pi, until the third passage. Samples were evaluated to check for SARS-CoV-2 replication, total RNA was extracted, and genomic and sg mRNA were tested using RT-qPCR, as described above.

### 2.5. Immunofluorescence Assay

Vero cells were seeded at 20,000 cells/well in 8-well chamber slides (Nunc Lab-TekChamber Slide System, Thermo Fisher Scientific, USA). Infected and control cell cultures were fixed in Ethanol 95% for 30 min at 4 °C, followed by 15 min at 28 °C and subsequently in Carson Millonig formalin [31] for 5 min at 25–30 °C. The cells were permeabilized for 30 min with 0.01% Triton X-100 (Merck, Rahway, NJ, USA). The slides were incubated at 37 °C for 1 h with a rabbit polyclonal anti-SARS-CoV-2 coronavirus Spike protein antibody (Subunit 1) (cat.PA5-81795, Thermo Fisher Scientific, USA). Secondary antibody AlexaFluor 488 conjugated goat anti-Rabbit (cat. A11008, Invitrogen, Waltham, MA, USA) was applied at 37 °C for 1 h, followed by counterstaining with 1:5000 DAPI (final concentration of 1 μg/mL; cat.03571, Molecular Probes, Eugene, OR, USA). All Slides were analyzed under a laser scanning microscope (LSM) 710 confocal microscope (ZEISS, Oberkochen, Germany).

### 2.6. SARS-CoV-2 Whole-Genome Sequencing

SARS-CoV-2 whole-genome amplification and sequencing were performed as previously described by Naveca et al. [32], using the Illumina COVIDSeq test kit or using in-house sequencing protocols [33]. The consensus sequences were generated using ViralFlow 0.0.6 [34]. The Pangolin algorithm assigned PANGO lineages to the obtained genome sequences [35]. Multiple sequence alignments were generated via MAFFT [36]. Single Nucleotide Polymorphisms (SNPs) presented against the high-quality reference sequence WIV04 (EPI_ISL_402124) were determined via the snipit (https://github.com/aineniamh/snipit (accessed on 14 October 2022)), and coverage was determined using ViralFlow 0.0.6, after the genome assembly. All genomic and epidemiological data associated with the NS, RS, and the cell-cultured virus from RS were uploaded to the EpiCoV database in the GISAID with the following accession numbers: EPI_ISL_15515006, EPI_ISL_15515007, and EPI_ISL_15515008, respectively.

### 2.7. Statistical Analysis

Statistical analyses were performed using GraphPad Prism software v8.4.1 (GraphPad Software, San Diego, CA, USA). The Mann–Whitney U test was used to assess the significant difference between SARS-CoV-2 cycle threshold (Ct) values obtained for N1, N2, and sg mRNA. A *p*-value < 0.05 was considered to be statistically significant.

## 3. Results and Discussion

We analyzed 1633 samples, consisting of 333 RS, 697 NS, and 603 saliva samples from 176 COVID-19 cases and contacts. Overall, 130 (73.9%) subjects had at least one sample that tested positive for SARS-CoV-2. The positivity rates for SARS-CoV-2 by testing NS, saliva, and RS samples were 50.2% (350/697), 28.4% (171/603), and 12.9% (43/333), respectively. Table 1 shows detailed information about epidemiological data and positivity rates by age group.

**Figure 1:**
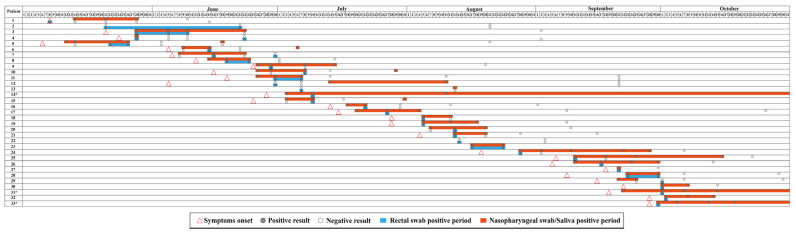
Detail information regarding SARS-CoV-2 dynamics in patients with positive RS samples. * Patients with SARS-CoV-2 positive NS samples in November.

For RS samples, we detected SARS-CoV-2 RNA in 43 samples from 33 participants, and from those, only two participants tested negative for SARS-CoV-2 using NS or saliva samples. Figure 1 shows detailed information regarding SARS-CoV-2 dynamics in participants with positive RS samples. Ct-values of positive-RS samples ranged from 25.2 to 36.4, with a median value of 33.5.

To confirm the presence of SARS-CoV-2 RNA, RS samples with high Ct-values (>30) were retested (RNA extraction and RT-qPCR detection), and the detection was confirmed in all positive-RS samples, with minor variations of Ct-values. Fecal shedding of SARS-CoV-2 RNA is an important sign of infection, with implications for public health and control of the disease. Several studies have reported the detection of SARS-CoV-2 RNA in fecal or RS samples from COVID-19-positive individuals, with detection rates ranging from 16.5% up to 90%, together with reports of prolonged viral shedding in stool specimens [37,38,39,40]. In a study involving hospitalized and non-hospitalized patients from Denmark, SARS-CoV-2 RNA was detected in RS samples of 53.8% (28/52) of COVID-19-positive adults and children, with virus detection of up to 45 days. The study found no significant difference in disease severity among hospitalized patients with positive or negative SARS-CoV-2 rectal detection [41]. Another study demonstrated SARS-CoV-2 fecal RNA shedding for up to seven months in cases of mild and moderate COVID-19 [42]. The authors also found a correlation between fecal viral RNA shedding and gastrointestinal symptoms in patients who had cleared their respiratory infection.

**Figure 2:**
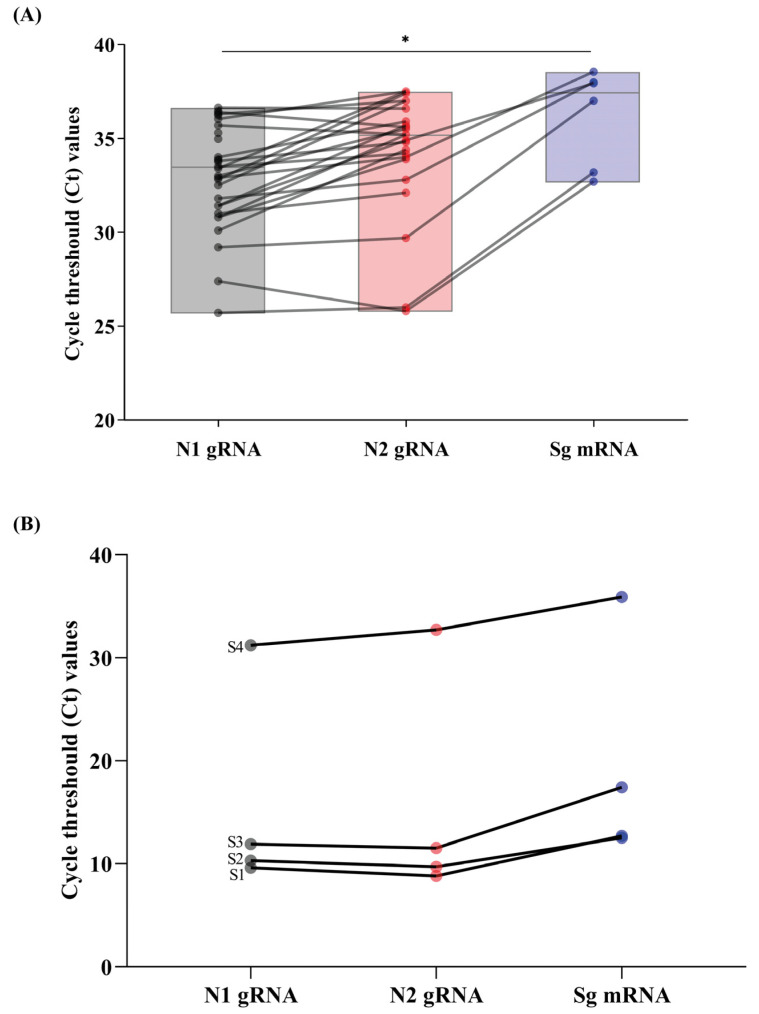
SARS-CoV-2 positive samples from rectal swab samples via RT-qPCR for N1, N2, and subgenomic message RNA (sg mRNA). (**A**) Box-plot with Ct values of SARS-CoV-2 positive samples and sg mRNA. Upper and lower grey horizontal lines indicate maximum and minimum values, respectively, and central gray horizontal lines indicate medians. * *p* < 0.05 (Mann–Whitney U-test). (**B**) Ct values of N1, N2, and sg mRNA of a rectal swab sample (S4), first passage (S3), second passage (S2), and cell culture supernatant containing previously isolated SARS-CoV-2 used as a positive control (S1).

Additionally, we investigated the positive RS samples for the presence of sgN mRNA. sgN mRNA was detected in 19.4% (6/31) of samples with Ct-values ranging from 33.4 to 38.6. It is worth noting that the Ct-values for sgN mRNA were higher (>5 cycles) compared to those found for N1 genomic RNA (*p* < 0.05) (Figure 2A).

While transmission of SARS-CoV-2 typically occurs from person to person through close contact, respiratory droplets, and aerosol particles [40,43,44,45,46], some studies have suggested the possibility of fecal-oral transmission [19,20,24,29,30,37]. It is well known that the SARS-CoV-2 cellular receptor (ACE2) is highly expressed and abundantly present in humans, including in lung alveolar epithelial cells and enterocytes of the small intestine [47], and many studies have detected the presence of SARS-CoV-2 RNA in feces and RS samples from infected individuals [39,40,41].

In this present study, we detected sgN mRNA from RS samples, which provides evidence of active SARS-CoV-2 replication in intestinal cells. As sg mRNA is transcribed only in infected cells and is not packaged into virions, its detection indicates the presence of actively infected cells in samples. Figure 2B shows evidence of replication and sg mRNA detection demonstrated by increasing viral load after viral isolation in cell culture of a clinical RS sample (S4), passage 1 (S3), passage 2 (S2), and positive control (S1).

Wölfel et al. [29] and Dimcheff et al. [48] have demonstrated the detection of sgN RNA from respiratory samples as an indicator of active SARS-CoV-2 replication, in addition to virus isolation in cell culture. Qian et al. [49] demonstrated directed evidence of active SARS-CoV-2 viral replication in the intestine of a COVID-19 patient during the incubation period of the disease. Viral RNA and sg mRNA detection via RT-PCR does not really imply the presence of an infectious virus in a fecal sample. Although evidence suggests that SARS-CoV-2 is capable of infecting and replicating in enterocytes [49,50], this does not necessarily imply the shedding of infectious SARS-CoV-2 from feces.

**Figure 3:**
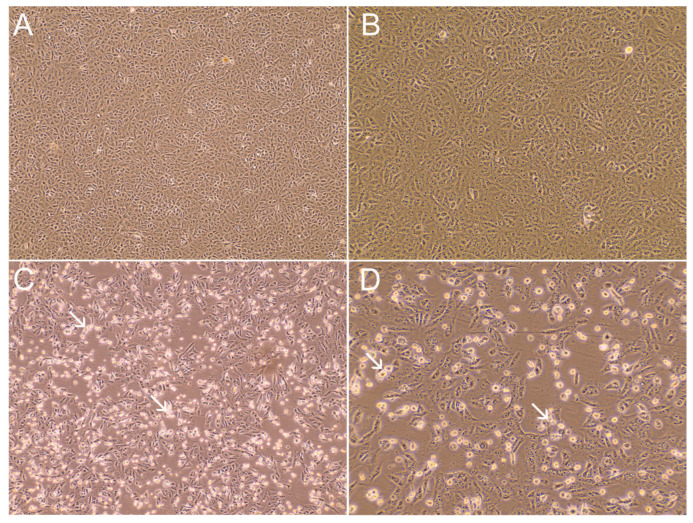
SARS-CoV-2 cytopathic effect (CPE) in Vero cells observed following virus infection MOI 0.02 at 48 hpi. (**A**) Mock-infected Vero cells. (**B**) Positive rectal swab without visible CPE (sample id_31386). (**C**) Positive rectal swab sample with CPE (sample id_30771). (**D**) SARS-CoV-2 passage 2 from sample id_30771. Representative areas of CPE are indicated by white arrows. Panels (**A**,**C**) are shown with 100× magnification; panels (**B**,**D**) are shown with 200× magnification.

**Figure 4:**
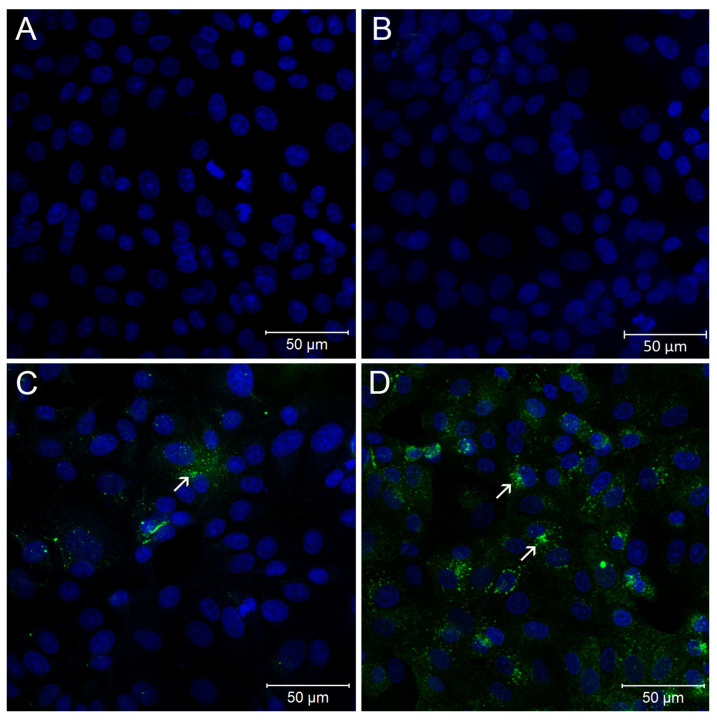
Immunofluorescence assay of Vero cells inoculated with rectal swab samples. (**A**) Mock-infected Vero cells. (**B**) Rectal swab sample (id_31386). (**C**) Rectal swab sample (id_30771). (**D**) Second passage of sample id_30771. Nucleus—Blue; SARS-CoV-2 Spike—Green. In MOCK infected and sample id_31386, staining for spike protein was not observed (**A**,**B**). Rectal swab sample (id_30771) positive staining for spike in cytoplasmic dots (**C**). Supernatant of second passage of the sample id_30771 showing the immunofluorescence in a higher number of cells (**D**). Representative areas of SARS-CoV-2 infected cells are indicated by white arrows.

To investigate the fecal shedding of infectious SARS-CoV-2, we inoculated 14 RS samples in cell culture with different Ct values (varying from 25.7 up to 38). We successfully isolated infectious SARS-CoV-2 from one RS sample (Figure 3 and Figure 4). The rectal swab sample (id_30771) was collected from a 68-year-old woman who tested positive for SARS-CoV-2, with a Ct value of 25.2. A cytopathic effect (CPE) was observed after 3 days of incubation, and the Ct value of the infected-culture supernatant was 9.1. A second-round passage (2P), performed three days post-infection, generated a viral stock (Ct value of 9.2, and 1.14 × 10^6^ PFU/mL) for additional SARS-CoV-2 characterization experiments. The remaining 13 RS samples did not show CPE after three passages. To confirm the absence of CPE in cell cultures, we tested via RT-qPCR, the genomic and sg RNA of all the supernatant samples collected from the passages, including mock and positive controls. None of the supernatants collected showed positive results for N1 and N2 genomic RNA or sgN mRNA, and samples were considered negative for SARS-CoV-2 infectiousness. COVID-19-positive NS samples, used as positive controls, inoculated in Vero cell culture showed CPE in 72 h after inoculation and tested positive for both genomic RNA and sgN mRNA detection, thus corroborating the usefulness of the molecular assay to demonstrate active viral replication.

**Figure 5:**
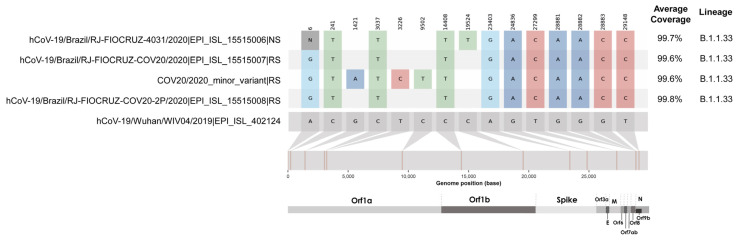
Analysis of substitutions present in nasopharyngeal swab (NS) (EPI ISL 15515006), rectal swab (RS) (major -EPI_ISL_15515007- and minor consensus -COV20/2020_minor_variant-) and viral isolate from RS sample (EPI_ISL_15515008).

The full-length viral genome sequence was obtained by next-generation sequencing from NS, RS, and isolated virus from clinical RS samples (Figure 5). Interestingly, we demonstrated by whole genome sequencing, a single nucleotide polymorphism in the SARS-CoV-2 genome sequence obtained from the NS sample compared to the sequence obtained from RS collected 7 days later. This mutation occurred in Orf1b:C19524T, specifically in the nsp15 gene, found in a frequency of 96.7% of reads and a depth in this region of 1123×. This type of substitution is indicative of the APOBEC-like host protein system that is frequent in SARS-CoV-2 [51]. Interestingly, only for the sample obtained from RS, we observed the presence of a minority variant through intra-host analysis. Viral subpopulations are associated with the host’s immune response, and the longer the patient is infected, the greater the intra-host complexity [52].

As found by Simmonds et al. [53], sequence substitutions in the SARS-CoV-2 genome were characterized by a preponderance of cytidine-to-uridine (C→U), also called transitions. The APOBEC3 gene family members that are primarily involved in antiviral defense show evidence of extensive positive selection and expansion over the course of mammalian evolution, particularly in the primate lineage. In the same line, Dezordi et al. [51] stated that the antiviral mechanism mediated by APOBEC-like host proteins may affect the identification of coinfection events based on the analysis of SARS-CoV-2 intra-host diversity. Both studies guided our findings that the patient in our study was infected by the B.1.1.33, and the host antiviral mechanism enabled the C→U transition. Interestingly, we recovered this major consensus in the NS collection, but this did not occur in the sample collected from RS. In addition, this phenomenon occurred at only one site in the genome comparing these two specimen sources. The minor variant found in the RS present transitions events as well, and the same substitution at Orf1a:C9502T. Despite its importance, it represents a small part of the patient’s viral load, unlike the sample collected from the NS. As evolutive impacts, the underlying mechanism of hypermutation also drives much of the amino acid sequence diversity observed in SARS-CoV-2, as well as an imbalance in frequencies of bases in the coronavirus genome [53].

As shown in Figure 5, most of the consensus sequences of NS, RS, and second-passage isolated virus showed high nucleotide identities, and it is possible to confirm that the isolation of the RS sample was efficient, presenting only the sequence of the majority consensus of the sample whose virus was isolated. The marked substitution at position 6 shows an “N” for the NS sample. In this case, this is not an informative site, as non-sequenced areas at the beginning and at the end of the sequence commonly occur in sequencing methodologies.

To our knowledge, few studies have been able to detect and isolate infectious SARS-CoV-2 from the fecal or rectal swab samples of infected individuals [20,54,55,56]. It is well established that SARS-CoV-2 RNA is commonly found in feces in COVID-19 patients. However, whether the excretion of infectious viruses is a common or a rare event remains unclear. Xiao et al. [20] demonstrated the detection of infectious SARS-CoV-2 from the feces of a unique patient who died of COVID-19. The Ct value of the fecal sample used was 20.8 (N gene), and visible CPE was observed in Vero cells 48 h after a second-round passage. Our study showed the presence of infectious SARS-CoV-2 from 1 out of 14 rectal swab samples inoculated in Vero cells. We believe that low viral titers observed in the other SARS-CoV-2 positive samples may explain the in vitro non-infectiousness results. Moreover, Zang et al. [50] showed that SARS-CoV-2 could be in vitro inactivated by simulated colonic fluid, suggesting that the virus may be rapidly inactivated when released to the intestinal lumen. Bullard et al. [57] demonstrated that SARS-CoV-2 Vero cell infectivity was only observed for nasopharyngeal swab samples showing Ct < 24. A previous study from our group demonstrated the detection of SARS-CoV-2 in stool samples from patients presenting only acute gastroenteritis symptoms and failed to isolate infectious viruses from stool samples [58].

Our study has several limitations. First, the logistic challenges of conducting home visits resulted in variable adherence to study procedures, missed visits, and delays in the enrollment of families. Second, we did not test fecal sample infectivity at different time points during infection nor used in vivo models. Third, viral culture is laborious and needed for special biosafety facilities (BSL3), which were available in reduced usage time due to other ongoing research projects. In addition, viral isolation from feces is technically challenging, and the 0.22 µm filtration treatment applied may reduce viral concentration in the original sample. Fourth, our study includes SARS-CoV-2 viruses before the emergence of any variants of concerns (VOCs); therefore, our results do not apply to VOCs with different viral fitness with regard to gut infection capacity and fecal excretion of viable viruses. Finally, more research into the infectivity pattern for RS samples of these VOCs under the same conditions applied in this study is required.

## 4. Conclusions

In conclusion, through sgN mRNA, we detected replicating SARS-CoV-2 in RS samples, and through cell culture, we found infectious SARS-CoV-2 particles from one RS sample from a COVID-19 patient. Some studies have failed to isolate SARS-CoV-2 from fecal samples or rectal swabs, regardless of viral RNA concentration in these samples [25,29,56]. Even though SARS-CoV-2 can be shed in feces for prolonged periods, often longer than in the respiratory tract samples [38,39], the contagiousness of SARS-CoV-2 from the fecal–oral route remains unknown. Additionally, our results have implications concerning SARS-CoV-2 detection in wastewater, raising the question of whether infectious viruses are present in those samples or whether the contact with raw sewage samples contributes to SARS-CoV-2 transmission remains to be definitively demonstrated.

## Figures and Tables

**Table 1:** Number of tested and SARS-CoV-2 positive samples from rectal swab, nasopharyngeal swab, and saliva samples from symptomatic SARS-CoV-2 individuals and contacts included in the study distributed by age group and gender.

Age Groups *	Participants (%)	Gender (%)		Sample Type—Positive/Tested (%)		
		Male	Female	Rectal Swab	Nasopharyngeal	Saliva
3 m–≤12 m	5 (2.8)	3 (60)	2 (40)	9/13 (69.2)	8/15 (53.3)	0/0 (0)
12 m–≤60 m	11 (6.3)	9 (91.8)	2 (18.2)	4/16 (25)	3/19 (15.8)	0/2 (0)
5 y–≤20 y	10 (5.7)	6 (60)	4 (40)	3/18 (16.6)	21/39 (53.8)	11/35 (31.4)
20 y–≤60 y	122 (69.3)	47 (38.5	75 (61.5)	23/232 (9.9)	275/515 (53.4)	141/468 (30.1)
≥60 y	28 (15.9)	10 (35.7)	18 (64.3)	4/54 (7.4)	43/109 (39.4)	19/98 (19.4)
Total	176 (100)	75 (42.6)	101 (57.4)	43/333 (12.9)	350/697 (50.2)	171/603 (28.4)

* Age groups are expressed in months (m) or years (y) old.

## Data Availability

The data presented in this study are openly available in the EpiCoV database in the GISAID with the following accession numbers: EPI_ISL_15515006, EPI_ISL_15515007, and EPI_ISL_15515008. This SARS-CoV-2 study is registered in the Brazilian National System for Genetic Heritage and Associated Traditional Knowledge Management (SisGen, No. A7DD919 and A2196ED).
